# Glycerin suppositories used prophylactically in premature infants (SUPP) trial: a study protocol for a pilot randomized controlled trial

**DOI:** 10.1186/s40814-015-0024-0

**Published:** 2015-08-25

**Authors:** Michael H. Livingston, Jorge Zequeira, Henrietta Blinder, Julia Pemberton, Connie Williams, J Mark Walton

**Affiliations:** 1 McMaster Pediatric Surgery Research Collaborative, McMaster Children’s Hospital, Health Sciences Centre Room 4E3, 1280 Main Street West, Hamilton, Ontario L8S 4K1 Canada; 2 Clinician Investigator Program, Postgraduate Medical Education, McMaster University, MDCL Rm 3101, 1280 Main Street West, Hamilton, Ontario L8S 4K1 Canada; 3 Division of Pediatric Surgery, University of Puerto Rico School of Medicine, University of Puerto Rico—Medical Sciences Campus, San Juan, 00921 Puerto Rico; 4 Division of Pediatric Surgery, Department of Surgery, McMaster University, Health Sciences Centre Room 4E3, 1280 Main Street West, Hamilton, Ontario L8S 4K1 Canada; 5 Division of Neonatology, Department of Pediatrics, McMaster University, Health Sciences Centre Room 4F5, 1280 Main Street West, Hamilton, Ontario L8S 4K1 Canada

**Keywords:** Prematurity, Newborn, Feeding intolerance, Suppository, Necrotizing enterocolitis

## Abstract

**Background:**

Feeding is a significant challenge for premature infants in the neonatal intensive care unit (NICU). These patients are often treated with glycerin suppositories to stimulate the passage of meconium and prevent feeding intolerance. Unfortunately, the evidence for this practice is inconclusive.

**Methods/design:**

This protocol is for an external pilot study that will assess the feasibility of a superiority, placebo-controlled, parallel-design, multicenter randomized controlled trial. Participants are premature infants treated in a level 3 NICU with a gestational age 24 to 32 weeks and/or birth weight of 500 to 1500 g. Thirty participants will be recruited as part of this external pilot study. Participants will be randomized to glycerin suppository (250 mg) or placebo starting 48 to 72 h after birth and continuing once daily until meconium evacuation is complete or for a maximum of 12 days. The placebo consists of a 250-mg glycerin suppository placed in the diaper rather than the rectum. Study treatments are administered by the charge nurse on duty who is not otherwise involved in patient care. All other clinicians and research personnel will remain blinded. Outcomes for the pilot study are percentage of eligible participants randomized, percentage of infants reaching full enteral feeds, cost, and treatment-related adverse events (rectal bleeding, rectal perforation, and anal fissure).

**Discussion:**

This external pilot study will assess the feasibility of a multicenter randomized controlled trial of glycerin suppositories in premature infants. The subsequent multicenter trial will have sufficient power to determine whether this treatment strategy is associated with decreased time to full enteral feeds.

**Trial registration:**

ClinicalTrials.gov: NCT02153606

## Background

Feeding is a significant challenge for premature infants in the neonatal intensive care unit (NICU) [1, 2]. These babies have immature digestive tracts and can develop a life-threatening bowel infection called necrotizing enterocolitis (NEC) [3, 4]. Treatment of this condition may require surgery and is associated with substantial morbidity and mortality. This includes short bowel syndrome, dependence on parenteral nutrition, and/or need for additional surgery [5, 6].

Infants who do not develop NEC can still have issues with feeding and growth. Most are supported with intravenous nutrition while enteral feeds are advanced over a period of 1 to 3 weeks. This process can be delayed if infants develop feeding intolerance, characterized by abdominal distension, undigested feeds in the stomach, and decreased bowel movements [7]. This can lead to increased reliance on intravenous nutrition, which is associated with sepsis, extrauterine growth restriction, and poor neurodevelopmental outcomes [1, 2, 8].

Glycerin suppositories are commonly used in premature infants to stimulate the passage of meconium and improve feeding tolerance [9]. This practice is based on the observation that preterm infants experience significant delays in the passage of meconium, which is more viscous than normal stool [10, 11]. Delays in meconium evacuation appear to be associated with a delay in the transition to enteral feeding [12]. Thus, if meconium evacuation could be expedited through the use of glycerin suppositories, this may lead to faster transition to enteral feeding, decreased reliance on intravenous nutrition, and better outcomes. Unfortunately, there is little evidence to support this practice [9–17].

We recently conducted a systematic review on the use of glycerin suppositories and enemas in premature infants [17]. We identified a total of 185 infants from three single-center, randomized controlled trials [14–16]. These studies focused on the prophylactic use of glycerin suppositories (two trials) or enemas (one trial). Across all three trials, there were no differences in terms of meconium evacuation, transition to full enteral feeding, or mortality. There were no reports of rectal bleeding or perforation, but meta-analyzed data revealed a non-significant trend towards increased risk of NEC with active treatment. We concluded that going trials should be carefully monitored and stopped if it becomes clear that this trend is a real effect and not just a spurious correlation.

The results of our systematic review were complicated by the fact that all three trials were underpowered and affected by one or more major methodological issues. As a result, the quality of evidence was low to very low. We concluded that the evidence for the use of glycerin suppositories or enemas in premature infants is inconclusive and that further research is required. As a result, we designed an external pilot study to assess the feasibility of a multicenter randomized controlled trial of prophylactic glycerin suppositories in premature infants.

## Methods/design

### Study design and objective

The glycerin suppositories used prophylactically in premature infants (SUPP) trial is an external pilot study for a superiority, placebo-controlled, parallel-design, multicenter randomized controlled trial [18]. The purpose of the multicenter trial is to determine whether glycerin suppositories decrease the time to full enteral feeding in premature infants. We hypothesize that the multicenter trial will demonstrate that using glycerin suppositories in premature infants results in earlier completion of meconium evacuation. Whether this treatment strategy results in earlier full enteral feeding or improvements in other outcomes remains unclear [17].

The study protocol described here is for an external pilot study to assess the feasibility of a multicenter randomized controlled trial [19, 20]. This includes assessments of cost, recruitment, protocol violations, post-randomization exclusions, and treatment-related adverse events. The pilot will also allow us to determine if modifications to the inclusion or exclusions criteria or duration of study treatments might be required in the multicenter trial.

### Setting

Premature infants will be recruited from the level 3 NICU at McMaster Children’s Hospital in Hamilton, Ontario, Canada. This unit treats almost 1000 infants per year of which approximately 150 would be eligible for our trial.

### Participants

The participants will be premature infants 24 to 32 weeks and/or birth weight 500 to 1500 g (Table 1). Exclusion criteria include the following: congenital gastrointestinal anomalies, surgery within 48 h of birth; culture-proven sepsis, vasopressors, nitric oxide, prostaglandins, suspected coagulopathy (bleeding from any orifice), confirmed coagulopathy (international normalized ratio >1.4, partial thromboplastin time >39 s, fibrinogen <1.00 g/L, platelet count <100 × 109/L), neutropenia (absolute neutrophil count <0.5 × 10^9^/L), and complete meconium evacuation within 48 h after birth.

**Table 1:** Participant inclusion and exclusion criteria

Criteria	Definition
Inclusion (any of the following)	
Gestational age	24–32 weeks gestation
Birth weight	500–1500 g
Exclusion (any of the following)	
Congenital gastrointestinal anomalies	Any congenital gastrointestinal anomalies
Clinically unwell	Major surgery within 48 h of birth
	Culture-proven sepsis
	Vasopressors
	Nitric oxide
	Prostaglandins
Suspected coagulopathy	Bleeding from any orifice
Confirmed coagulopathy	International normalized ratio >1.4
	Partial thromboplastin time >39 s
	Fibrinogen <1.00 g/L
	Platelet count <100 × 10^9^/L
Neutropenia	Absolute neutrophil count <0.5 × 10^9^/L
Complete meconium evacuation	2 bowel movements with no meconium

### Interventions

**Fig. 1:**
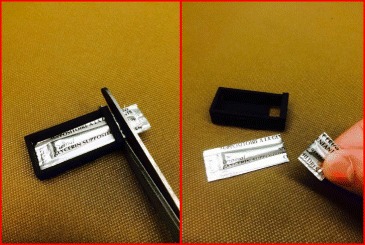
Plastic measurement guide to ensure consistent dose of glycerin suppositories

Participants randomized to active treatment will receive a 250-mg glycerin suppository once daily starting 48 to 72 hours after birth (i.e., on day 3 of life). This smaller suppository will be created by cutting the tip off of a 1440 mg glycerin suppository. In order to maintain consistent dosing, we created a plastic measurement guide that results in a 250-mg suppository (Fig. 1). This “tip” will be covered with a water-based lubricant and placed in the infant’s rectum.

Participants in the control group will receive placebo suppositories. In usual practice, partially dissolved suppositories are often ejected from the rectum either with or without stool. In our trial, leaving a suppository in the diaper (but not in the rectum) makes it ambiguous as to whether it was placed in the rectum and ejected or simply placed in the diaper. This approach also ensures that treatment will appear to have been administered to all infants, even if they happen to be in the control group.

Following administration of either treatment, the gluteal buttocks will be held together for 30 s to minimize the likelihood of the suppository being ejected from the rectum. Participants in the each treatment group will receive study treatments once daily until they pass two normal bowel movements free of meconium staining. A similar duration of treatment was used in the randomized controlled trial of glycerin enemas from Austria [14]. Maximum treatment duration will be 12 days (i.e., all treatments will stop on day 14 of life).

All study interventions will be administered by one of the NICU charge nurses on duty. These individuals have years of experience working in the NICU but are not involved in the care of individual patients. All participants will receive a medical order of “nil per rectum” during the period of study treatments. This will be removed once study treatments stop. Study participants will be eligible for rescue glycerin suppository therapy if they are judged by the medical team to have feeding intolerance.

### Randomization

**Fig. 2:**
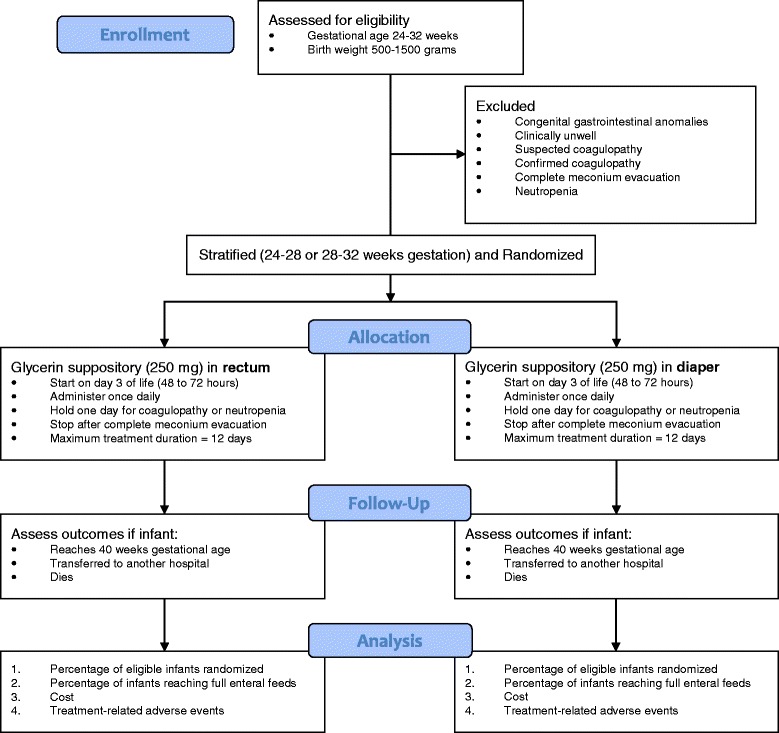
Overview of glycerin suppositories used prophylactically in premature infants (SUPP) trial

Infants will be allocated to treatment groups via web-based stratified blocked randomization. Previous studies have shown that the size of the infant is highly predictive of the time to full enteral feeds [13]. In order to maintain prognostic balance, participants will be stratified by gestational age: (1) 24–27 weeks 6 days; or (2) 28–31 weeks 6 days. This strategy has been used in other randomized controlled trials of feeding intolerance in premature infants [14, 15, 21, 22]. See Fig. 2 for an overview of the external pilot study.

Sequence generation will be created using random number software by an unblinded research assistant not otherwise involved in the SUPP Trial. Block size will range from four to six to ensure that there are an equal number of participants in the treatment and control groups. Randomization of study participants will be completed online by a blinded research assistant immediately prior to the administration of the first study treatment. This web-based system will be created using Research Electronic Data Capture (REDCap) software [23]. For each participant, REDCap will assign a three-digit treatment code that will be recorded by the blinded research assistant on the infant’s bedside chart. The charge nurse will use a coding sheet to link the three-digit number for each participant with either active treatment or placebo.

### Blinding

The principal investigator, co-investigators, parents or guardians, physicians, bedside nurses, allied health professionals (e.g., dieticians), research assistants, outcome assessors, and statistical analysts will be blinded to treatment allocation. The only groups who will be unblinded are the participants (i.e., the premature infants who cannot communicate) and the charge nurses responsible for administering study treatments. Adequate blinding of medical staff, bedside nurses, and allied health professionals is essential since these clinicians typically make decisions about advancing or holding feeds during daily patient rounds. If these individuals are aware which treatment the participant is receiving, there is a chance that this knowledge will affect their decision-making and bias the results.

We will employ several strategies to maintain blinding. First, participants in the control group will receive suppositories placed in the diaper rather than no intervention at all. Second, all study treatments will be administered when the participant’s crib is covered. This will ensure that only the charge nurse administering the treatment knows which intervention is given. Third, we have held multiple meetings with the NICU nursing staff prior to the start of the trial to discuss the purpose of blinding and ensure that we have adequate buy-in from these individuals.

### Duration of treatment

Infants enrolled in this trial will receive active treatment or placebo once daily until meconium evacuation is complete. This will be defined as two bowel movements free of meconium staining. Previous studies have demonstrated that this process takes approximately 6 to 9 days [14]. The study intervention will be withheld and reassessed daily in cases of thrombocytopenia (platelet count < 100 × 10^9^/L), suspected coagulopathy (bleeding from any orifice, confirmed coagulopathy (international normalized ratio >1.4, partial thromboplastin time >39 s, fibrinogen <1.00 g/L, thrombocytopenia (platelet count <100 × 10^9^/L), or neutropenia (absolute neutrophil count <0.5×10^9^/L).

The study intervention will be stopped early in cases of clinical deterioration (vasopressors, prostaglandins, culture-proven sepsis, NEC, or death) or treatment-related adverse events (rectal bleeding, rectal perforation, or anal fissure). Previous trials of glycerin suppositories and enemas did not report single case of rectal bleeding, rectal perforation, or anal fissure [14–16]. As such, the risk of these events occurring in the current study is low.

### Follow-up

Outcome data will be extracted from the medical record after participants are discharged from hospital, reach a corrected gestational age of 40 weeks or die (whichever happens first). The research assistant will also assess participants on at least a weekly basis to maintain contact with nursing staff, discuss any protocol violations, monitor for adverse events, and address any other issues that arise. Nursing staff will also be instructed to submit possible adverse events to the research assistant as soon as they occur. These include any cases of rectal bleeding, rectal perforation, and anal fissure.

### Outcomes

Outcomes for the external pilot study will be recruitment rate (i.e., percentage of eligible infants randomized), completion rate (i.e., percentage of infants reaching the primary endpoint of full enteral feeds), and treatment-related adverse events (i.e., safety outcomes). The pilot study will also allow us to better estimate the explicit cost per infant of conducting a randomized trial on this topic (i.e., considering the total costs of database design, data storage, printed materials, and salary for research assistants). We will also assess the frequency and type of protocol violations and post-randomization exclusions.

The primary outcome for the multicenter trial will be time in days to full enteral feeding (defined as 150 mL/kg/day). Advancing the rate of enteral feeds is typically based on a standardized NICU feeding protocol [24–26]. Deviations from this protocol occur when infants become unwell, develop signs of feeding intolerance, or if there are other clinical concerns. Secondary outcomes will include feeding volume on day 14 of life (in mL/kg), days to complete meconium evacuation, days of parenteral nutrition, incidence of NEC, incidence of line sepsis, compliance with treatment regimen, and mortality.

### Sample size

For the external pilot study, we hope to recruit 30 participants (15 per group) over a 6-month period. Previous reviews recommend using at least 12 participants per group, and some indicated that a total sample size of 30 may be more appropriate [27–29].

### Potential pitfalls

Adherence to the study protocol was a serious issue in a randomized controlled trial of glycerin enemas from Austria [14]. This may have occurred because some clinicians did not believe in the efficacy of glycerin enemas and tended to withhold this intervention among participants in the therapeutic arm. This will be less of an issue in our trial since glycerin suppositories are commonly used in the NICU at McMaster Children’s Hospital and are much less invasive.

Another issue to consider is post-randomization withdrawals or exclusions. While this is an issue in any randomized controlled trial, this will present a unique challenge in our study since participants will have been alive for less than 48 h when they are enrolled. Some infants may not have had prenatal screening and serious congenital anomalies that would have excluded them from the study may not be diagnosed until days or weeks after randomization. Even in cases of excellent prenatal care, some conditions (e.g., Hirschsprung’s disease) cannot be diagnosed until the postnatal period [30, 31]. The best way to handle this will be to follow all randomized participants to the primary endpoint of full enteral feeds and analyze the data on the basis of intention-to-treat.

Losing participants to follow-up is unlikely to occur since all premature infants are monitored in hospital until they are tolerating full enteral feeds and reach 37 weeks corrected gestational age. Some participants who are doing well clinically may be transferred to a NICU in a community hospital that is not part of this study, but this is unlikely to occur until after these infants have reached full enteral feeds. Finally, the mortality rate in this population is approximately 10 % [32, 33]. While our exclusion criteria will exclude most of the infants at risk for postnatal mortality, some participants may die before they are randomized, complete the treatment regimen, or reach full enteral feeds.

### Statistical analysis

Frequencies and 95 % confidence intervals using normal approximation will be used to estimate recruitment rate and percentage infants reaching the primary endpoint. We will also report the rate of protocol violations, post-randomization exclusions, and incidence of adverse events related to the study intervention. We will also report the mean cost per infant randomized. All data will be analyzed in the Statistics Package for the Social Sciences (SPSS) [34].

### Ethical and safety considerations

This study was approved by the Neonatal Research Committee at McMaster Children’s Hospital, Hamilton Integrated Research Ethics Board (14–575), and Health Canada (9427-M1133-53C). All parents or guardians will provide written and informed consent prior to enrollment.

We have established a Data Safety and Monitoring Board (DSMB) for the pilot study, which consists of two neonatologists and one pediatric surgeon. This group will meet after the first five participants are randomized and then once every 3 months until the pilot study is complete. The DSMB will review safety outcomes (i.e., treatment-related adverse events) and can request unblinding should the need arise. Unblinding will be facilitated by the unblinded research assistant who performed sequence generation and is not otherwise involved in the administration of this study. The principal investigator, co-investigators, and research assistant will remain blinded.

## Discussion

The evidence for the use of glycerin suppositories in premature infants is inconclusive [9, 17]. In our recent systematic review, we considered the results from three single-center randomized controlled trials of glycerin suppositories (two studies) or enemas (one study) [17]. The trial focused on glycerin enemas included 81 very low birth weight infants from a single hospital in Austria [14]. All participants were enrolled in the study shortly after birth and stratified by gestational age either (1) 24–27 weeks 6 days or (2) 28–31 weeks 6 days. Infants in the intervention group received daily glycerin enemas if they did not pass meconium spontaneously within 12 h of birth. These enemas continued until complete evacuation was achieved. The control group did not receive any intervention.

This study was an open trial, the primary outcome was the number of days to complete evacuation of meconium, and the study was powered to detect a 30 % difference. There was a trend towards a treatment effect with complete evacuation of meconium occurring at a median of 6.5 days in the intervention group and 9 days in the control group, but this difference was not statistically significant (*p* = 0.11). No differences were reported for any of the secondary outcomes, including duration of hospital stay, weight at discharge, days to introduction of oral feedings, feeding volume on day 14 of life, days to passage of first meconium, or days to full enteral feeding. This trial was limited by the lack of blinding, possibility of selective reporting, small sample size, and frequent protocol violations.

The second trial explored whether glycerin suppositories decrease feeding intolerance in premature infants [15]. Participants were enrolled shortly after birth and were randomized from a stack of opaque envelopes. This study was an open trial, and there were no attempts to maintain blinding. The primary outcome was days to full enteral feeding. There was a trend towards a decrease in time to full feeds of 1.6 days, but the study was only powered to detect a difference of 3.6 days. There were also no significant differences for any of the secondary outcomes, including incidence of NEC, episodes of culture-positive sepsis, feeding intolerance during the first 10 days, growth and nutrition, and ventilation. Despite this, infants in the intervention group passed their first stool earlier (day 2) than controls (day 4) (*p* = 0.016) and were less likely to pass their first stool after 48 h of life (24 versus 64 %) (*p* = 0.003).

The third randomized controlled trial was published in 2014 [16]. This study included 50 premature infants from a single hospital in India with a gestational age of 28 to 32 weeks and birth weight 1000 to 1500 g. Infants less than 28 weeks gestation or 1000 g were excluded. Participants randomized to active treatment received a 1000 mg glycerin suppository once daily starting on day 2 of life and continuing until day 14, regardless of stooling pattern. Infants in the control group underwent a placebo procedure, where the diaper was opened and closed again, but no active treatment was administered. All study treatments were administered by a research nurse, and blinding was maintained for all other clinical and research personnel.

This trial reported no differences between treatment groups for any of the outcomes, including time to full enteral feeds, time to regain birth weight, NEC, frequency of feeds being withheld, and length of hospital stay. The main limitations were small sample size, possibility of selective reporting, and number of participants lost to follow-up. In each group, 3/25 participants (greater than 10 % of the total sample size) were transferred to another hospital before complete outcomes could be obtained [16].

As shown above, previous trials on the use of glycerin suppositories in premature infants are small, underpowered, and affected by a variety of methodological issues [17]. As such, the evidence for this treatment strategy is inconclusive and clinical equipoise remains. The SUPP trial will start as an external pilot study to assess feasibility. If minimal changes are required, we will develop a similar protocol for a superiority, placebo-controlled, parallel-design, multicenter randomized controlled trial. Once completed, the multicenter trial will have sufficient power to determine whether glycerin suppositories facilitate meconium evacuation and transition to enteral feeding in premature infants.

## Trial status

The SUPP trial started recruiting participants in January 2015 and is on track to complete enrollment of 30 participants for the external pilot study by July 2015. An update with results from the external pilot study will be provided in 2016.

## Abbreviations

NEC: necrotizing enterocolitis
NICU: neonatal intensive care unit
